# Functional cooperation between IK_Ca_ and TRPC1 channels regulates serum‐induced vascular smooth muscle cell proliferation via mediating Ca^2+^ influx and ERK1/2 activation

**DOI:** 10.1111/cpr.13385

**Published:** 2022-12-23

**Authors:** Xiaoling Jia, Xinlan Chen, Chao Gao, Haikun Wang, Chengxi Yang, Lin‐Hua Jiang, Yubo Fan

**Affiliations:** ^1^ Key Laboratory for Biomechanics and Mechanobiology of Ministry of Education, Beijing Advanced Innovation Center for Biomedical Engineering, School of Biological Science and Medical Engineering, and with the School of Engineering Medicine Beihang University Beijing China; ^2^ Sino‐UK Joint Laboratory of Brain Function and Injury of Henan Province, and Department of Physiology and Pathophysiology Xinxiang Medical University Xinxiang China; ^3^ A4245‐Transplantation, Immunology and Inflammation, Faculty of Medicine University of Tours Tours France; ^4^ School of Biomedical Sciences, Faculty of Biological Sciences University of Leeds Leeds UK

## Abstract

The increased proliferation of vascular smooth muscle cells (VSMCs) contributes to the pathogenesis of vascular diseases. The intermediate conductance calcium‐activated potassium (IK_Ca_) channel plays a critical role in VSMC proliferation by raising the intracellular calcium concentration ([Ca^2+^]_i_), but the underlying mechanism is still not unclear. Here we investigated the cooperation between IK_Ca_ and transient receptor potential canonical 1 (TRPC1) channels in mediating extracellular Ca^2+^ entry, which in turn activates downstream Ca^2+^ signalling in the regulation of VSMC proliferation using serum‐induced cell proliferation model. Serum‐induced cell proliferation was accompanied with up‐regulation of IK_Ca_ expression and an increase in [Ca^2+^]_i_. Serum‐induced cell proliferation and increase in [Ca^2+^]_i_ were suppressed by IK_Ca_ inhibition with TRAM‐34 or IK_Ca_ knockdown. Serum‐induced cell proliferation was strongly reduced by the removal of extracellular Ca^2+^ with EGTA or intracellular Ca^2+^ with BAPTA‐AM and, additionally, by TRPC1 knockdown. Moreover, the increase in [Ca^2+^]_i_ induced by serum or by IK_Ca_ activation with 1‐EBIO was attenuated by TRPC1 knockdown. Finally, serum induced ERK1/2 activation, which was attenuated by treatment with TRAM‐34 or BAPTA‐AM, as well as TRPC1 knockdown. Consistently, serum‐induced cell proliferation was suppressed by ERK1/2 inhibition with PD98059. Taken together, these results suggest that the IK_Ca_ and TRPC1 channels cooperate in mediating Ca^2+^ influx that activates the ERK1/2 pathway to promote cell proliferation, thus providing new mechanistic insights into VSMC proliferation.

## Graphical abstract

Schematic summary of proposed mechanisms for serum‐induced VSMC proliferation. The IK_Ca_ and TRPC1 channels cooperate to mediate Ca^2+^ influx and an increase in [Ca^2+^]_i_ that in turn activates the ERK pathway and thereby stimulates VSMC proliferation.

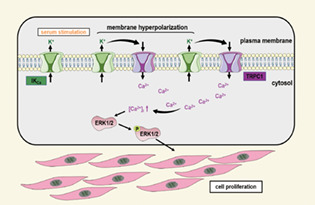

## 1 INTRODUCTION

Vascular smooth muscle cells (VSMCs) constitute to form the media of the blood vessel wall and play a pivotal role in maintaining vasculature structure and function.^1^ Unlike other mature cell types in adult body, VSMCs exhibit remarkable plasticity in their function. In normal arteries, VSMCs adopt a contractile phenotype to regulate vascular tone and blood pressure.^1^, ^2^, ^3^ In response to environmental changes, VSMCs switch to a synthetic phenotype, and proliferate excessively and migrate into blood vessel lumen. It has been well documented that the proliferative functionality of VSMCs contributes to the pathogenesis of multiple vascular diseases, such as hypertension, atherosclerosis and restenosis.^4^, ^5^, ^6^, ^7^, ^8^, ^9^

Ion channels are strongly implicated in the regulation of VSMC function and pathogenesis of related vascular diseases.^10^, ^11^, ^12^, ^13^ The intermediate conductance Ca^2+^‐activated K^+^ (IK_Ca_) channel, also known as K_Ca_3.1 or IKI encoded by the KCNN4 gene, is highly expressed in synthetic VSMCs, and is critically engaged in VSMC proliferation.^14^, ^15^, ^16^, ^17^, ^18^ Compelling evidence exists to show that VSMC proliferation induced by chemical or physical stimuli is accompanied with increased expression and function of the IK_Ca_ channel and that its pharmacological and/or genetic interventions effectively decrease VSMC proliferation and alleviate the progression of diseases, such as atherosclerosis and restenosis.^19^, ^20^, ^21^ Despite its well‐documented role in VSMC proliferation, the signalling mechanism by which the IK_Ca_ channel partakes in VSMC proliferation remains not well understood.

Several studies have hypothesized that membrane hyperpolarization induced by activation of the IK_Ca_ channel provides a driving force for extracellular Ca^2+^ entry, leading to an increase in [Ca^2+^]_i_ and subsequent activation of Ca^2+^‐dependent downstream signalling pathways,^19^ but the supporting evidence is still awaited. Specifically, this hypothesis raises two interesting and important questions: which Ca^2+^ entry mechanism is regulated by the IK_Ca_ channel and what is the specific downstream Ca^2+^‐dependent signalling pathway that is involved in VSMC proliferation?

It has been reported that the expression of Ca^2+^‐permeable transient receptor potential canonical (TRPC) channels is increased in synthetic VSMCs and vascular diseases.^12^, ^22^, ^23^, ^24^ In particular, the TRPC1 expression was up‐regulated in balloon‐induced human internal mammary arteries^25^ and also in human vein samples after angioplasty.^26^ Importantly, treatment with inhibitory anti‐TRPC1 antibodies prevented neointima progression in rodents.^26^ Furthermore, there is evidence that TRPC1 interacts with Orai1 and Homer to mediate store‐operated Ca^2+^ entry (SOCE) and promotes VSMC proliferation and migration, contributing to neointima formation and stenosis in balloon‐injured carotid arteries in rats.^27^ Evidence also exists that the TRPC1 channel mediates extracellular Ca^2+^ entry induced by activation of the IK_Ca_ channel in breast cancer cells.^28^ However, the interplay between the IK_Ca_ and TRPC1 channels in modulating the [Ca^2+^]_i_ in VSMCs and its role in cell proliferation are not established. Ca^2+^ is a universal second messenger and controls a wide range of cellular processes including cell proliferation^11^, ^29^, ^30^, ^31^ via diverse Ca^2+^‐dependent signalling pathways, including ERK, PKA and Akt/PI_3_K,^32^, ^33^, ^34^, ^35^ and ERK is one of the most common mechanisms in the regulation of VSMC proliferation.^33^, ^36^, ^37^, ^38^, ^39^

In this study, we investigated the interplay between the IK_Ca_ and TRPC1 channels in raising the [Ca^2+^]_i_ and the Ca^2+^‐dependent downstream signalling pathways in serum‐induced VSMC proliferation. Our results demonstrated that activation of the IK_Ca_ channel promotes Ca^2+^ entry through the TRPC1 channel, leading to an increase in [Ca^2+^]_i_ and subsequent activation of ERK1/2 to promote VSMC proliferation. Such a finding provides a mechanistic insight into VSMC proliferation and pathogenesis of related vascular diseases.

## 2 MATERIALS AND METHODS

### 2.1 Isolation and culture of VSMCs

All experiments related to use of animals were approved by the Animal Research Ethics Committee of Beihang University (SNU‐200916‐6‐1). VSMCs were isolated from the thoracic aortas of 1‐month‐old male Sprague–Dawley rats using enzymatic digestion and confirmed by the expression of α‐smooth muscle actin (α‐SMA) and smooth muscle myosin heavy chain (SM‐MHC), as previously described.^40^ VSMCs were cultured in Dulbecco's modified Eagle's medium (DMEM) (#210569010, Gibco) containing 10% foetal bovine serum (FBS, #11011‐8611, Sijiqing, China), 100 U/ml penicillin and 100 μg/ml streptomycin (#SV30010, Hyclone) at 37°C in a 5% CO_2_ incubator. VSMCs of passage 6–8 were used for the study.

### 2.2 SiRNA transfection

TRPC1‐specific siRNA (siTRPC1) (sense sequence 5′‐GCUUUUCUUGCUGGCGUGCdTdT‐3′; antisense sequence 5′‐GCACGCCAGCAAGAAAAGCdTdT‐3′), IK_Ca_‐specific siRNA (siIK_Ca_) (sense sequence 5′‐GGAGGUCCAGCUGUUCAUGdTdT‐3′; antisense sequence 5′‐CAUGAACAGCUGGACCUCCdTdT‐3′) and control siRNA (siCTL) (sense sequence 5′‐CUCGUUGUGCUUGCGUCUGdTdT‐3′; antisense sequence 5′‐CAGACGCAAGCACAACGAGdTdT‐3′), were custom‐designed and synthesized by Genepharma (Shanghai, China). VSMCs were seeded on 24‐well plates at a density of 2.5 × 10^4^ cells/well and grown in DMEM containing 10% FBS for 24 h, and then in DMEM without FBS (serum‐free medium) for further 24 h. Transfection with siRNA was performed using Lipofectamine™ 3000 (#L3000001, Thermo Fisher scientific) according to the manufacturer's protocols. 25 pmol siRNA and 1.25 μl Lipofectamine™ 3000 diluted with 125 μl opti‐MEM (#31985070, Thermo Fisher) were used in each well of cells. After incubated in transfection medium for 8 h, cells were maintained in DMEM containing 10% FBS for 48 h before they were used to examine the efficiency of knocking down protein expression using western blotting, cell proliferation using the MTT assay, and the [Ca^2+^]_i_ using single cell confocal imaging, as detailed below. The transfection efficiency was determined by transfection of cells with Cy3‐labelled siRNA, followed by single cell imaging of Cy3 fluorescence using a fluorescence microscope (Olympus, IX71), and 90% cells were Cy3 positive (Figure S1).

### 2.3 MTT assay

Cell proliferation was assessed using the MTT assay. 5 × 10^3^ cells were seeded in 96‐well plates and incubated overnight in DMEM containing 10% FBS, with three wells of cells prepared for each conditions in each independent experiment. Cells were then starved for 24 h in FBS‐free medium before cultured in DMEM without or with 10% FBS for further 48 h. In some experiments, cells were transfected with siRNA as described above. In some experiments, cells were treated with 100 nM TRAM‐34 (#T‐105, Alomone), 2 mM EGTA (#E4378, Sigma–Aldrich), 30 μM BAPTA‐AM (#M4973, AbMole), or 5 μM PD98059 (#167869‐21‐8, Santa Cruz) for 48 h, starting at the end of starvation.

Cells in each well were incubated with 100 μl 5 mg/ml MTT solution for 4 h at 37°C. After the removal of the MTT solution, 100 μl dimethyl sulfoxide (DMSO, #sc‐202581, Santa Cruz) added to each well to dissolve the purple formazon crystals, and the absorbance at 490 nm was measured using a multifunctional plate reader (Thermo Fisher).

### 2.4 Western blotting

Cells were seeded on 24‐well plates at a density of 2.5 × 10^4^ cells/well and cultured in FBS‐free medium for 24 h and then in medium containing 10% FBS for 48 h for examining the expression of IK_Ca_ and TRPC1, 5 min to 24 h for examining ERK1/2 activation, or 30 min for examining the effects of BAPTA‐AM on ERK1/2 activation. Treatment with 100 nM TRRAM‐34 started in last 30 min when cells were cultured in FBS‐free medium and continued 30 min in FBS‐containing medium. In some experiments using cells prior transfected with siRNA as described above, cells were cultured in FBS‐free medium for 24 h and then in medium containing 10% FBS for 30 min.

After above‐described treatments, cells were lysed in RIPA buffer (#P0013B, Beyotime, China) containing 1% protease inhibitor (#ST507, Beyotime, China), and protein concentration was determined using a bicinchoninic acid assay kit (#P0010S, Beyotime, China). Twenty micrograms proteins were separated in 10% SDS‐PAGE and transferred to PVDF membrane (#FFP26, Beyotime, China). Membranes were blocked with 5% bovine serum albumin (BSA, #ST025, Beyotime, China) in Tris‐buffered saline and Tween 20 (TBST, #ST673, Beyotime, China) for 1 h and then incubated with a primary antibody overnight at 4°C. The following primary antibodies were used: mouse anti‐IK_Ca_ antibody (1:100 dilution, #sc‐365265, Santa Cruz), mouse anti‐TRPC1 antibody (1:200 dilution, #sc‐133076, Santa Cruz), mouse anti‐t‐ERK1/2 or p‐ERK1/2 antibody (1:100 dilution, #sc‐514302 and sc‐136521; Santa Cruz), mouse anti‐PCNA antibody (1:500 dilution, #BM0104, Boster, China) and rabbit anti‐GAPDH antibody (1:500 dilution, #AB‐P‐R 001, Hangzhou Xianzhi Biotechnology, China). Membranes, after washed with TBST three times, each for 5 min, were incubated with a secondary horseradish peroxidase‐conjugated anti‐mouse IgG or anti‐rabbit IgG antibody (1:5000 dilution, Origene) for 1 h at room temperature. After extensively washed in TBST, protein bands were visualized using enhanced chemiluminescence (Sage Creation, SmartChemi ECL) and images captured by MiniChemi (Sagecreation) and analysed with ImageJ. GAPDH was used as loading control.

### 2.5 Calcium imaging

The change in [Ca^2+^]_i_ was monitored by single cell calcium imaging as previously described.^41^ Briefly, 5 × 10^4^ cells were seeded on 29‐mm glass‐bottom dishes and cultured in DMEM medium with or without 10% FBS for 48 h. In some experiments, cells were cultured in medium also containing 100 nM TRAM‐34. After that, cells were washed twice with Ca^2+^‐containing buffer (10 mM HEPES, 140 mM NaCl, 2 mM CaCl_2_, 1.13 mM MgCl_2_, 4.7 mM KCl, 10 mM Glucose), and loaded with 5 μM Fluo 4‐AM (#F14201, Thermo Fisher) for 1 h at room temperature. After washed with loading buffer twice, cells were maintained in Ca^2+^‐containing buffer. Images of fluorescence excited at 488 nm and emitted at 515 nm ~10 random fields were captured using a confocal microscope (Andor, Dragonfly). In experiments with siRNA‐transfected cells, cells were cultured 48 h in DMEM medium with or without 10% FBS. After loaded with Fluo 4‐AM dye described above, cells were maintained in Ca^2+^‐containing buffer or Ca^2+^‐free buffer. Fluorescence was recorded using a confocal microscopy (Andor, Dragonfly) at 500 ms intervals for 3 min. After 30 min recording to establish the basal level, 200 μM 1‐EBIO (#ab120258, Abcam) was added into Ca^2+^‐containing buffer, or 2 mM CaCl_2_ was added into Ca^2+^‐free buffer. The fluorescence intensity was analysed with the ImageJ.

### 2.6 Determination of membrane potential

Membrane potential was measured by imaging of DiBAC4(3), a fluorescence indicator of membrane potential. Cells transfected with siCTL, siIK_Ca_ or siTRPC1 were incubated with 200 nM DiBAC_4_(3) (#70363‐83‐6, Sigma–Aldrich) for 30 min at 37°C. Fluorescence was excited by 488 nm and emitted at 515 nm, and images were captured at 3‐s intervals for 1 min using an inverted fluorescence microscope (Olympus, IX71). 1‐EBIO (200 μM, Abcam) was added into the medium after 30 s. Fluorescence intensity was analysed with the ImageJ.

### 2.7 Data presentation and statistical analysis

All data are presented as means ± SD, where appropriate. Western blotting and MTT assays were performed at least three times, and Fluo4 fluorescence intensity was examined in approximately 100 cells. Student's *t*‐test was performed for comparison of two groups, and one‐way analysis of variance (ANOVA) followed by post hoc Fisher's test for comparison of three or more groups, with *p* < 0.05 being considered significant.

## 3 RESULTS

### 3.1 The IK_Ca_ channel mediates serum‐induced VSMC proliferation via raising the [Ca^2+^]_i_

**FIGURE 1:**
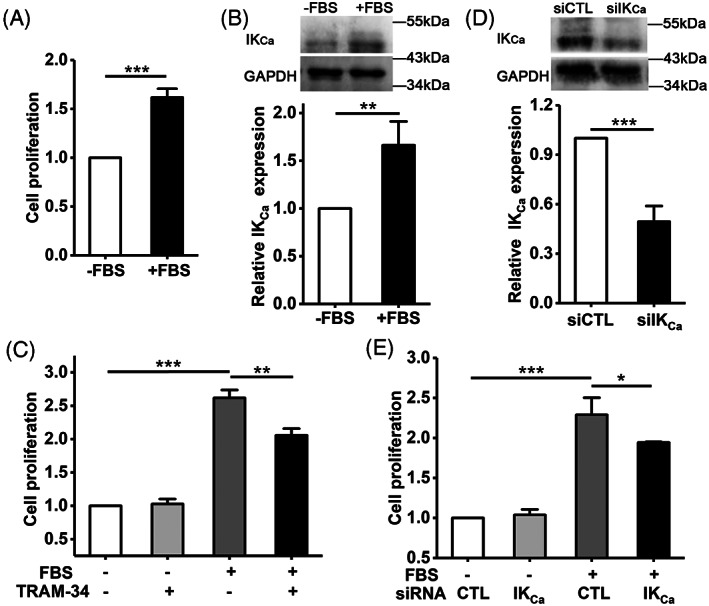
IK_Ca_ channel mediates serum‐induced VSMC proliferation. (A) Summary of cell proliferation in FBS‐free or FBS‐containing medium for 48 h, determined using the MTT assay, from three independent experiments. (B) Representative western blots (top) and summary of data from three independent experiments (bottom) showing the IK_Ca_ expression in cells after cultured in FBS‐free or FBS‐containing medium for 48 h. (C) Summary of cell proliferation in FBS‐free or FBS‐containing medium without or with 100 nM TRAM‐34 for 48 h, from three independent experiments. (D) Representative western blots (top) and summary of data from three independent experiments (bottom) showing the IK_Ca_ expression in cells, transfected with IK_Ca_‐specific siRNA (siIK_Ca_) or control siRNA (siCTL) and cultured in FBS‐containing medium for 48 h. (E) Summary of cell proliferation in cells, transfected with siIK_Ca_ or siCTL and cultured in FBS‐free or FBS‐containing medium for 48 h, from three independent experiments. Cell proliferation (A, C, E) or IK_Ca_ expression (B, D) is presented relative to that in cells cultured in FBS‐free medium (A, B), FBS‐free medium without TRAM‐34 (C), FBS‐containing medium with siCTL transfection (D), or FBS‐free medium without siRNA transfection (E). Data are presented in mean ± SD. *p < 0.05; **p < 0.01; ***p < 0.001

We first validated serum‐induced VSMC proliferation. As shown in Figure 1A, cell proliferation, determined using the MTT assay, was significantly faster when cells were cultured in FBS‐containing medium compared to cells in FBS‐free medium. FBS also induced a significant increase in the expression level of PCNA (Figure S2), a protein marker of cell proliferation, supporting FBS‐induced stimulation of cell proliferation. Interestingly, FBS‐induced cell proliferation was accompanied with up‐regulation of the IK_Ca_ protein expression, as revealed by western blotting (Figure 1B). To assess the role of the IK_Ca_ channel in FBS‐induced cell proliferation, cells were treated with TRAM‐34, an IK_Ca_ channel‐specific blocker. Treatment with 100 nM TRAM‐34 moderately but not completely reduced cell proliferation in the presence of FBS, with no significant effect on cell proliferation in the absence of FBS (Figure 1C). Similarly, siRNA‐mediated knockdown of the IK_Ca_ expression (Figure 1D) moderately attenuated FBS‐induced cell proliferation, again without effect on cell proliferation in the absence of FBS (Figure 1E). Taken together, these results indicate that the IK_Ca_ channel plays an important role in serum‐induced VSMC proliferation.

**FIGURE 2:**
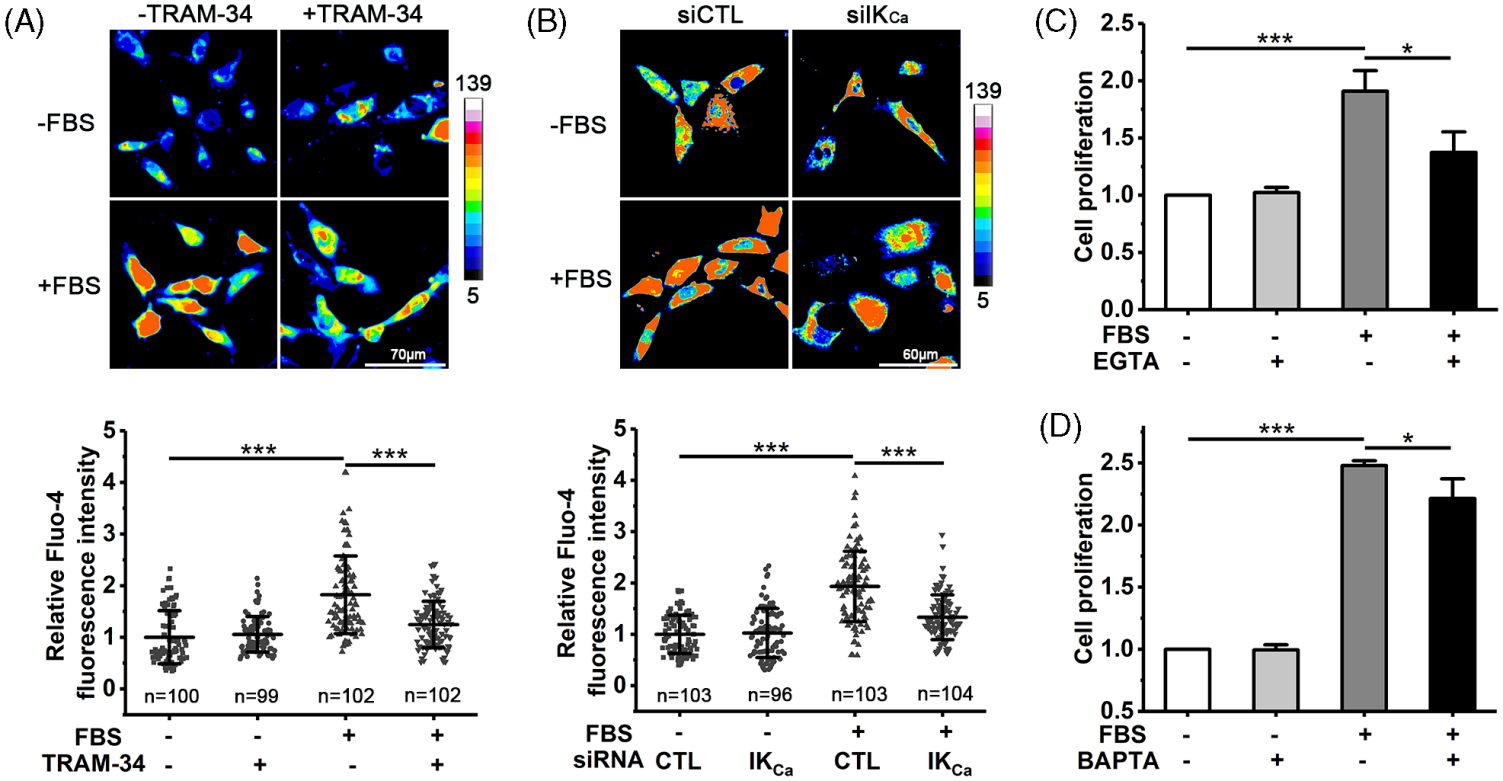
IK_Ca_ channel‐dependent increase in [Ca^2+^]_i_ is critical for serum‐induced VSMC proliferation. (A) Representative confocal images (top) and summary of relative changes in Fluo‐4 fluorescence intensity (indicative of [Ca^2+^]_i_) in indicated number of individual cells (bottom), cultured in FBS‐free or FBS‐containing medium without or with treatment with 100 nM TRAM‐34 for 48 h. (B) Representative confocal images (top) and summary of relative changes in Fluo‐4 fluorescence intensity in indicated number of individual cells (bottom), transfected with siRNA and cultured in FBS‐free or FBS‐containing medium for 48 h. Fluo‐4 fluorescence intensity was expressed relative to the mean fluorescence intensity in cells cultured in FBS‐free medium without TRAM‐34 (A) or with siCTL transfection (B). (C, D) Summary of cell proliferation determined using the MTT assay, in cells cultured in FBS‐free and FBS‐containing medium without or with 2 mM EGTA from three independent experiments (C), or with 30 μM BAPTA‐AM from three independent experiments (D). Cell proliferation is presented relative to that in cells cultured in FBS‐free medium without EGTA (C) or BAPTA‐AM (D). Data are presented in mean ± SD. *p < 0.05; ***p < 0.001

As introduced, the IK_Ca_ channel has been proposed to regulate cell proliferation through raising the [Ca^2+^]_i_. We next investigated whether it is the case in serum‐induced VSMC proliferation by combining confocal imaging and Fluo‐4, with an increase in its fluorescence intensity indicative of an increase in the [Ca^2+^]_i_. Exposure to serum induced an increase in [Ca^2+^]_i_ in individual cells, and such intracellular Ca^2+^ response was strongly suppressed by treatment with 100 nM TRAM‐34 (Figure 2A) and also by siRNA‐mediated knockdown of the IK_Ca_ expression (Figure 2B), consistently supporting a critical role of the IK_Ca_ channel in serum‐induced increase in [Ca^2+^]_i_. We further investigated the role of such Ca^2+^ signalling in FBS‐induced cell proliferation. Cell proliferation was significantly attenuated by addition of 2 mM EGTA in culture medium to remove extracellular Ca^2+^ (Figure 2C) or by treatment with 30 μM BAPTA‐AM to chelate intracellular Ca^2+^ (Figure 2D), strongly indicating that the increased [Ca^2+^]_i_ is critical in driving FBS‐induced cell proliferation. Collectively, these results suggest that the IK_Ca_ channel mediates serum‐induced VSMC proliferation via raising the [Ca^2+^]_i_.

### 3.2 The IK_Ca_ channel mediates serum‐induced cell proliferation via promoting TRPC1‐mediated Ca^2+^ entry

**FIGURE 3:**
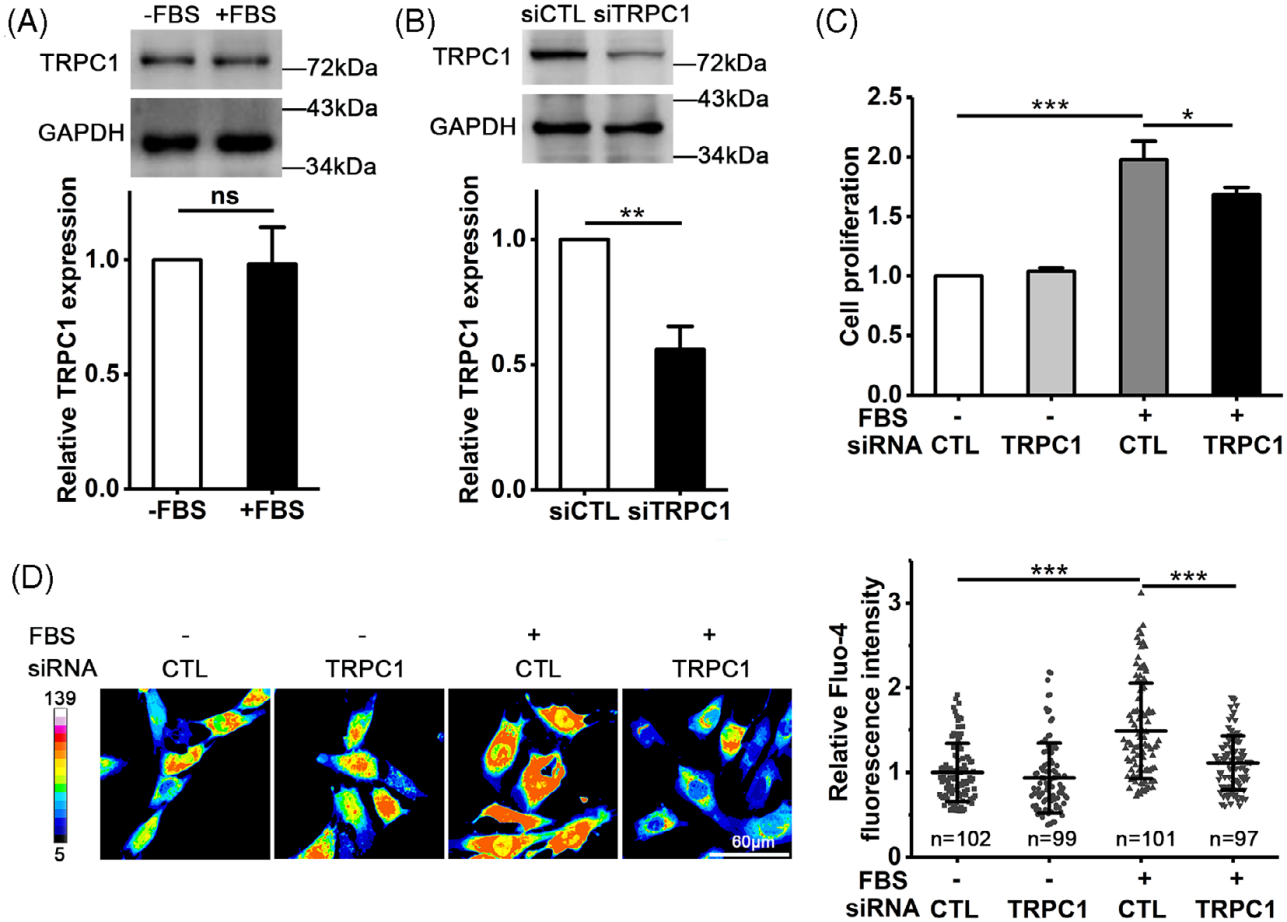
TPRC1 regulates serum‐induced VSMC proliferation and rise in [Ca^2+^]_i_. (A) Representative western blots (top) and summary of data from three independent experiments (bottom) showing that the TRPC1 expression in cells cultured in FBS‐free or FBS‐containing medium for 48 h. (B) Representative western blots (top) and summary of the data from three independent experiments (bottom) showing the TRPC1 expression in cells, transfected with TRPC1‐specific siRNA (siTRPC1) or control siRNA (siCTL) and cultured in FBS‐containing medium. (C) Summary of cell proliferation determined by the MTT assay in cells, transfected with siRNA and cultured in FBS‐free or FBS‐containing medium for 48 h, from three independent experiments. Cell proliferation is presented relative to that in cells cultured in FBS‐free medium with siCTL transfection. (D) Representative confocal images (left) and summary of relative changes in Fluo‐4 fluorescence intensity (indicative of [Ca^2+^]_i_) in indicated number of cells (right), transfected with siRNA and cultured in FBS‐free or FBS‐containing medium. Data are presented as mean ± SD. *p < 0.05; **p < 0.01; ***p < 0.001

The TRPC1 channel is known to play an important role in regulating cell proliferation via mediating Ca^2+^ entry^24^, ^27^ and therefore we were interested in whether the TRPC1 channel mediates serum‐induced cell proliferation as well as serum‐induced increase in [Ca^2+^]_i_. The TPRC1 expression in VSMCs was demonstrated by western blotting, with a similar expression level in cells cultured in FBS‐containing and FBS‐free medium (Figure 3A). SiRNA‐mediated knockdown of the TRPC1 expression, which was validated by western blotting (Figure 3B), reduced cell proliferation in the presence of FBS, but did not affect cell proliferation in the absence of FBS (Figure 3C). Furthermore, siRNA‐mediated knockdown of the TRPC1 expression strongly reduced FBS‐induced rise in [Ca^2+^]_i_ (Figure 3D). Taken together, these results clearly show that the TRPC1 channel in VSMCs is critically engaged in FBS‐induced increase in [Ca^2+^]_i_, as well as cell proliferation.

**FIGURE 4:**
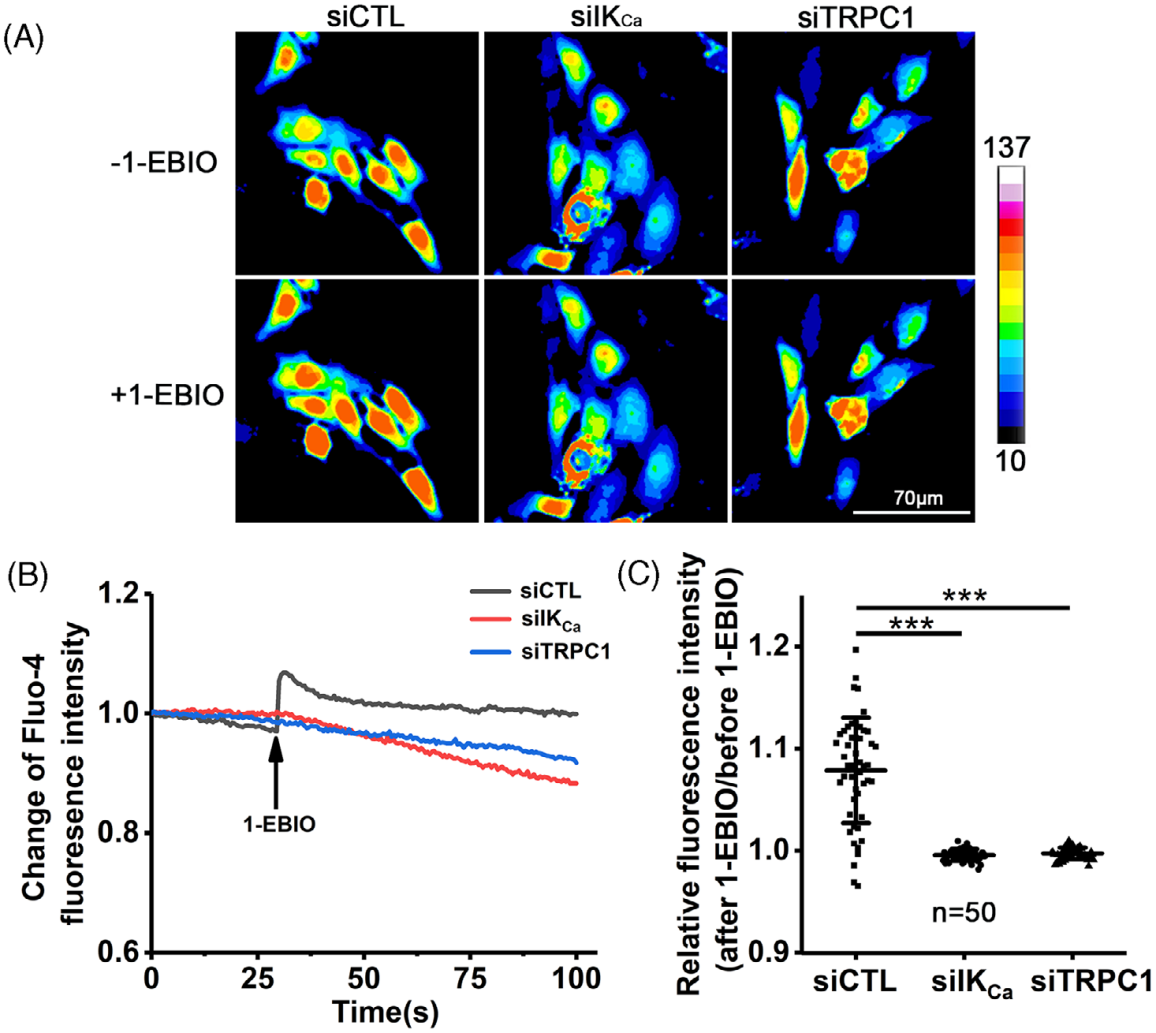
TRPC1 channel mediates IK_Ca_ activation‐induced rise in [Ca^2+^]_i_ in VSMCs. (A, B) Representative confocal images (A) and representative time‐course showing the change of Fluo‐4 fluorescence (indicative of [Ca^2+^]_i_) in one cell for each condition relative to the basal level (B). Cells were transfected with indicated siRNA respectively, and cultured in FBS‐containing medium for 48 h. Images were captured at 500 ms intervals, and 200 μM 1‐EBIO was added into the medium at 30 s (B). (C) Summary of relative changes in Fluo‐4 fluorescence intensity in indicated number of individual cells. Data are presented as mean ± SD. ***p < 0.001

**FIGURE 5:**
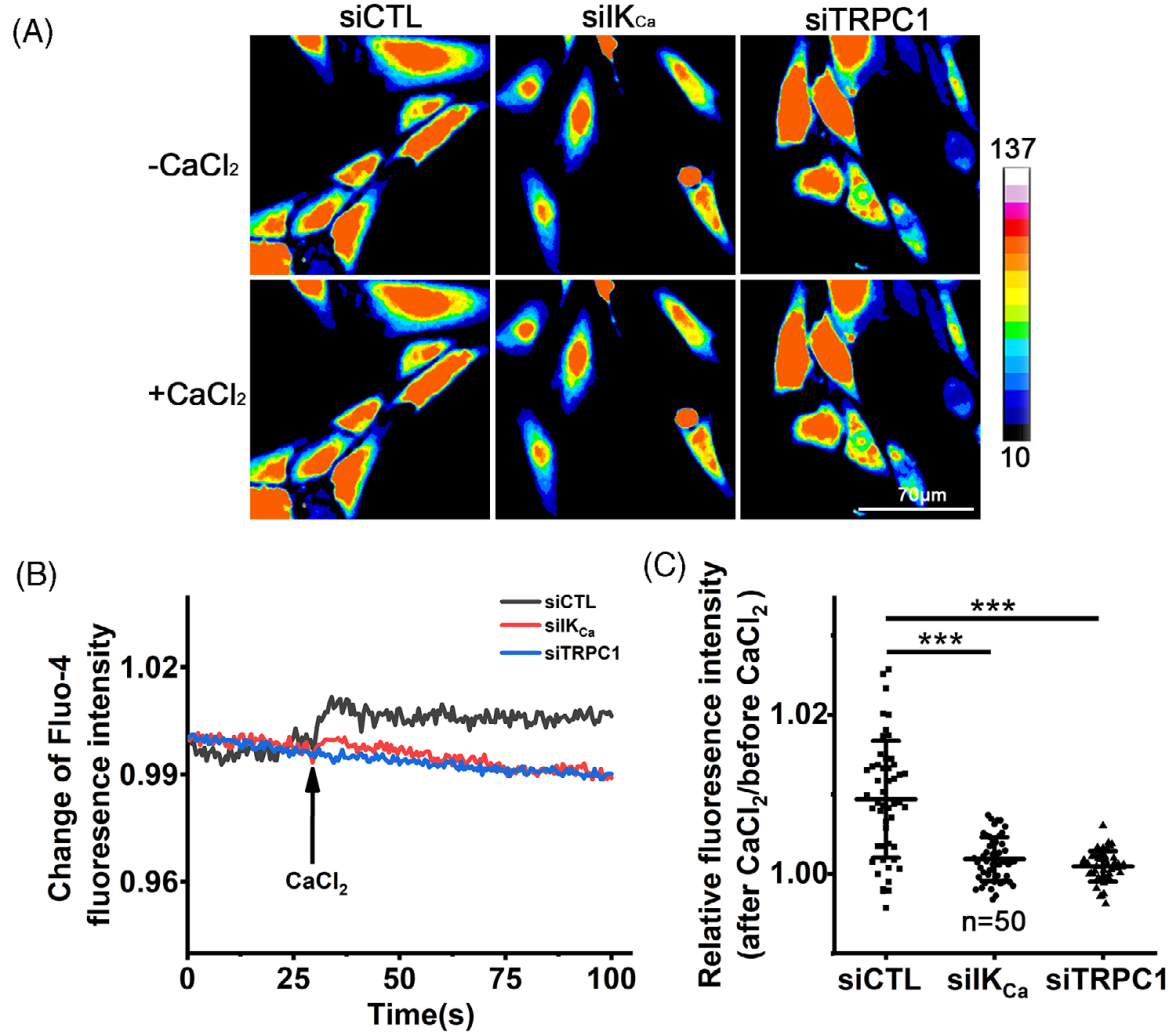
TRPC1 channel mediates serum‐induced Ca^2+^ entry regulated by IK_Ca_. (A, B) Representative confocal images (A) and representative time‐course showing the change in Fluo‐4 fluorescence intensity (indicative of [Ca^2+^]_i_) in one cells for each condition relative to the basal level upon addition of extracellular Ca^2+^ (B). Cells were transfected with indicated siRNA respectively, and cultured in FBS‐containing medium for 48 h. Images were captured at 500 ms intervals and 2 mM CaCl_2_ added into medium at 30 s. (C) Summary of relative changes in Fluo‐4 fluorescence intensity in indicated number of cells. Data are presented as mean ± SD. ***p < 0.001

To further investigate the relationship of the IK_Ca_ and TRPC1 channels in coordinating serum‐induced increase in [Ca^2+^]_i_, we used confocal imaging to monitor the Ca^2+^ responses to exposure to activation of the IK_Ca_ channel induced by 200 μM 1‐EBIO in individual cells that were prior transfected with siCTL, siIK_Ca_ or siTRPC1 (Figure 4). Exposure to 1‐EBIO resulted in a strong and sustained increase in [Ca^2+^]_i_ in siCTL‐transfected cells, but not in siIK_Ca_‐transfected cells. Similarly, there was little or no Ca^2+^ response to 1‐EBIO in siTRPC1‐transfected cells. To ascertain such an increase in [Ca^2+^]_i_ results from extracellular Ca^2+^ entry, we examined using a confocal microscopy (Figure 5) the Ca^2+^ responses in these cells that were initially maintained in Ca^2+^‐free solutions and subsequently introduced to Ca^2+^‐containing solutions. Introduction of extracellular Ca^2+^ led to a significant increase in [Ca^2+^]_i_ in siCTL‐transfected cells, but such Ca^2+^ response was largely absent in cells transfected with siIK_Ca_ or siTRPC1 (Figure 5). Collectively, these results clearly suggest that the IK_Ca_ channel raises the [Ca^2+^]_i_ in VSMCs through TRPC1‐mediated Ca^2+^ entry.

### 3.3 Serum‐induced Ca^2+^ signalling coordinated by the TRPC1 and IK_Ca_ channels drives VSMC proliferation via activating the ERK1/2 pathway

**FIGURE 6:**
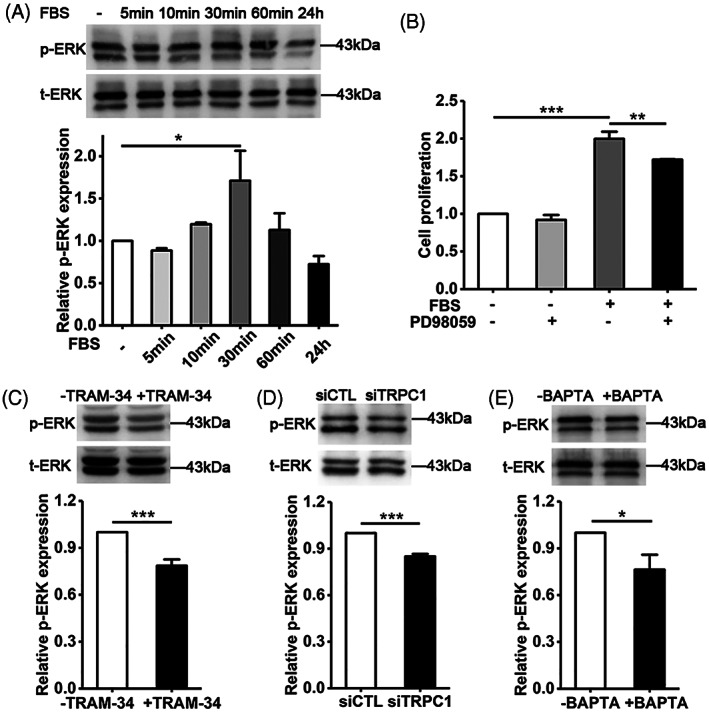
Serum‐induced Ca^2+^ signalling coordinated by IK_Ca_ and TRPC1 channels activates ERK to stimulate VSMC proliferation. (A) Representative western blots (top) and summary of the data from three independent experiments (bottom) showing phosphorylated and total ERK protein levels (p‐ERK and t‐ERK) in cells, cultured in FBS‐free medium for 24 h and then in FBS‐containing medium for indicated durations. (B) Summary of cell proliferation determined by the MTT assay in cells, cultured in FBS‐free medium for 24 h and FBS‐containing medium for 48 h with or without treatment with 5 μM PD98059, from three experiments. (C–E) Representative western blots (top) and summary of the data from three independent experiments (bottom) showing p‐ERK and t‐ERK levels in cells, treated with 100 nM TRRAM‐34 (C) or 30 μM BAPTA‐AM (D) in FBS‐free medium for 30 min and in FBS‐containing medium for further 30 min, cultured in FBS‐containing medium for 30 min after 48 h siRNA transfection (D). Cell proliferation is presented relative to that in cells in FBS‐free medium without PD98059 (B), and p‐ERK level relative to that in cells cultured in FBS‐free medium (A), without TRAM‐34 (C), with siCTL transfection (D) or without BAPTA‐AM (E). Data are presented as mean ± SD. *p < 0.05; **p < 0.01; ***p < 0.001

Finally, we investigated the Ca^2+^‐dependent downstream signalling pathway that transduces serum‐induced increase in [Ca^2+^]_i_ coordinated by the TRPC1 and IK_Ca_ channels to VSMC proliferation. ERK, as introduced above, represents one of the most common Ca^2+^‐dependent downstream signalling pathway in regulating VSMC proliferation. Indeed, exposure to FBS induced ERK activation indicated by a significant increase in its phosphorylated form shown in western blotting (Figure 6A). Interestingly, such ERK activation was highly dynamic, reaching the maximal level at 30 min and declining upon prolonged stimulation (Figure 6A). Serum‐induced VSMC proliferation, examined using the MTT assay (Figure 6B) or western blotting to compare the PCNA expression level (Figure S3), was suppressed by treatment with PD98059, an ERK inhibitor, indicating a critical role of the ERK pathway in mediating serum‐induced VSMC proliferation. To further support the IK_Ca_‐TRPC1‐Ca^2+^‐ERK signalling axis, we determined the effects of treatment with TRAM‐34, siRNA‐mediated knockdown of TRPC1 or treatment with BAPTA‐AM on FBS‐induced ERK activation. FBS‐induced ERK1/2 activation was significantly inhibited by treatment with 100 nM TRAM‐34 (Figure 6C), siRNA‐mediated knockdown of TRPC1 (Figure 6D), or treatment with 30 μM BAPTA‐AM (Figure 6E), indicating that serum‐induced increase in [Ca^2+^]_i_ coordinated by the IK_Ca_ and TRPC1 channels stimulates VSMC proliferation via activating the ERK1/2 pathway.

## 4 DISCUSSION

**FIGURE 7:**
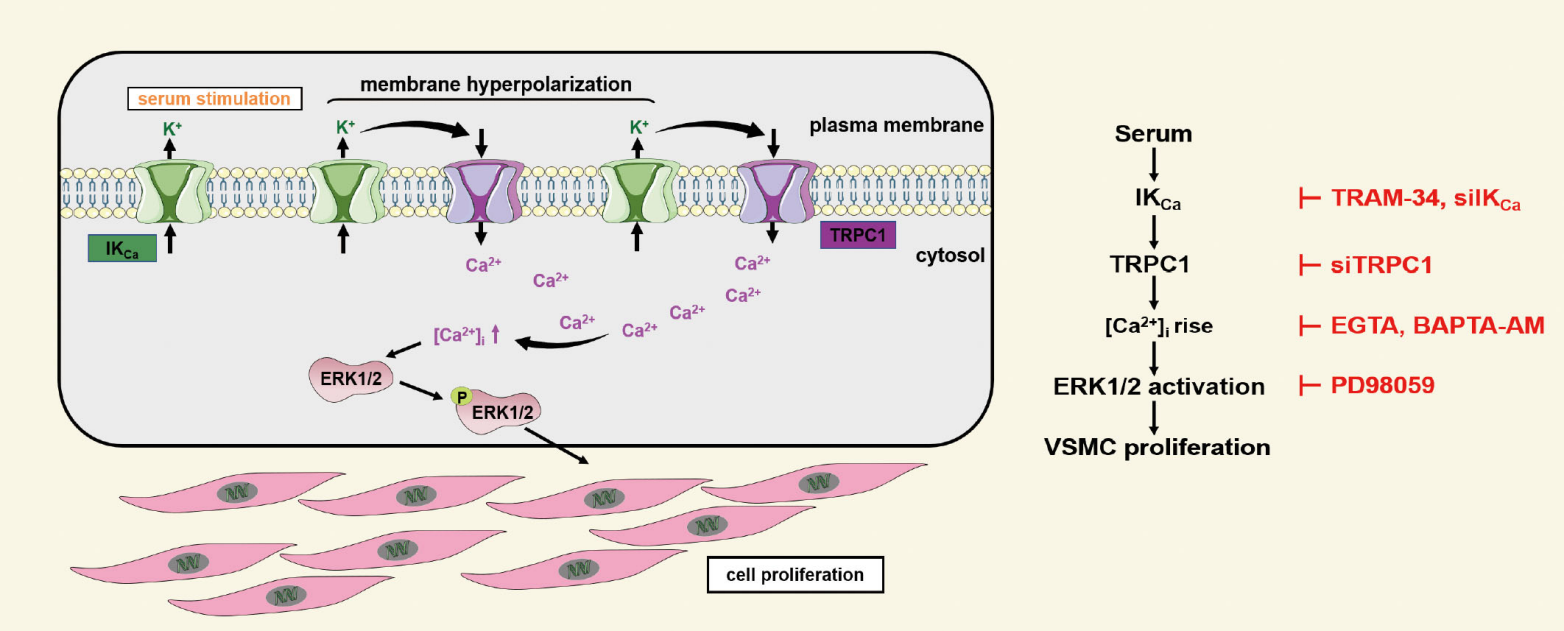
Schematic summary of proposed mechanisms for serum‐induced VSMC proliferation. The signalling molecules that activated or inhibited by the activators and inhibitors used in this study are highlighted in green and red, respectively.

The IK_Ca_ channel in VSMCs has been well known for its important role in regulating cell proliferation, a functional phenotype strongly implicated in the pathogenesis of multiple vascular diseases, but the underlying signalling mechanism is incompletely understood. In this study, we provide evidence to suggest that the IK_Ca_ channel cooperates with the TRPC1 channel to raise the [Ca^2+^]_i_ that in turn activates the ERK1/2 signalling pathway to drive serum‐induced VSMC proliferation (Figure 7).

In the present study, we demonstrated the importance of the IK_Ca_ channel expressed in VSMCs in serum‐induced cell proliferation via raising the [Ca^2+^]_i_ (Figures 1 and 2). The IK_Ca_ channel has been hypothesized, albeit being still short of supporting evidence, to increase the [Ca^2+^]_i_ via mediating K^+^ efflux to induce membrane hyperpolarization and thereby drive Ca^2+^ influx.^10^, ^15^, ^42^, ^43^ Such a hypothesis is highly consistent with membrane hyperpolarization upon 1‐EBIO‐induced activation of the IK_Ca_ channel in VSMCs (Figure S4).

Accumulating evidence indicates that up‐regulated TRPC1 expression is associated with increased cell proliferation and the pathogenesis of various vascular diseases, including atherosclerosis, pulmonary hypertension and restenosis, as well as lung cancer.^25^, ^26^, ^44^, ^45^ The present study showed that the TRPC1 expression remained unchanged during serum‐induced VSMC proliferation (Figure 3A) but genetic depletion of the TRPC1 expression reduced serum‐induced VSMC proliferation (Figure 3C) and increase in [Ca^2+^]_i_ (Figure 3D). These results strongly indicate that the TRPC1 channel is important in regulating serum‐induced Ca^2+^ signalling and cell proliferation. Nonetheless, further investigation is required to understand how the TRPC1 channel is activated during this process. The TRPC1 channel is widely viewed to regulate SOCE or act as a constituent of the mechanism mediating SOCE. Importantly, in this study, we provide strong evidence to support that the IK_Ca_ channel cooperates with the TRPC1 channel expressed in VSMCs to raise the [Ca^2+^]_i_ or more specifically promotes TRPC1‐mediated Ca^2+^ entry (Figures 3, 4, 5). A similar molecular mechanism has been proposed in the regulation of breast cancer cell proliferation.^28^ These findings clearly extend our understanding of the mechanisms engaging the IK_Ca_ and TRPC1 channels in regulating cell proliferation. One interesting and important question that arises and needs further research is whether the IK_Ca_ and TRPC1 channel interact with each other to form a signalling complex to coordinate serum‐induced Ca^2+^ signalling that stimulates VSMC proliferation.

In this study, we have further shown the importance of IK_Ca_/TRPC1‐coordinated Ca^2+^‐dependent activation of the ERK signal pathway in driving serum‐induced cell proliferation. We have recently reported a similar mechanism by which the IK_Ca_ channel mediates stiff substrate‐induced Ca^2+^ signalling and cell proliferation in VSMCs.^41^ Interestingly, our study revealed serum‐induced ERK activation was highly dynamic, declining and even lost upon prolonged exposure to FBS (Figure 6A). This fast kinetic of ERK activation is consistent with the fast kinetic of Ca^2+^ response to serum, which shows a fast initial strong Ca^2+^ response followed by a relative steady Ca^2+^ response (Figure S5). We showed that serum‐induced ERK activation in VSMCs was inhibited by treatment with TRAM‐34 to block the IK_Ca_ channel (Figure 6C), siRNA‐mediated knockdown of TRPC1 (Figure 6D) and, importantly, by treatment with BAPTA‐AM to chelate intracellular Ca^2+^ increase (Figure 6E). Collectively, these results are strong in supporting the concept that the IK_Ca_ and TRPC1 channels coordinate serum‐induced Ca^2+^ signalling that in turn activates the ERK pathway and thereby stimulates VSMC proliferation (Figure 7).

## 5 CONCLUSIONS

In summary, our present study shows that the IK_Ca_ channel cooperates with the TRPC1 channel in serum‐induced VSMC proliferation, via promoting TRPC1‐mediated Ca^2+^ entry to raise the [Ca^2+^]_i_ that in turns activates the ERK signalling pathway. Such a finding provides novel insights into the role of the IK_Ca_ and TRPC1 channels in VSMC proliferation and the pathogenesis of related cardiovascular diseases.

## AUTHOR CONTRIBUTIONS

Xiaoling Jia, Xinlan Chen, Lin‐Hua Jiang and Yubo Fan were responsible for the experimental design, data analyses and manuscript preparation. Xinlan Chen conducted most of the experiments. Chao Gao, Haikun Wang and Chengxi Yang helped with sample collection and analyses. Xiaoling Jia and Yubo Fan were responsible for project administration. All authors approved the submitted version.

## CONFLICT OF INTEREST

The authors declare that they have no known competing financial interests or personal relationships that could have appeared to influence the work reported in this article.

## Supporting information


**Figure S1.** The representative image showing the siRNA‐transfected cells. siRNA was labelled with Cy3 (red colour). The percentage of red‐stained cells suggests that almost a majority of cells were successfully transfected.
**Figure S2.** Serum induced an increase in the PCNA expression in VSMC. Representative western blots (top) and summary of data from three experiments (bottom) showing PCNA expression in cells cultured in the absence and presence of 10% FBS. **p < 0.01.
**Figure S3.** ERK inhibition with PD98059 decreased the PCNA expression. Representative western blots (top) and summary of data from three experiments (bottom) showing PCNA expression in cells cultured in presence of 10% FBS without or with treatment with 5 μM PD98059. **p < 0.01.
**Figure S4.** IK_Ca_ channel activation by 1‐EBIO results in membrane hyperpolarization in VSMC. (a) Representative recordings showing addition of 1‐EBIO induced an increase in DiBAC4(3) fluorescence intensity in cells transfected with siIK_Ca_, but not in cells transfected with siCTL or siTRPC1. (b) Summary of relative DiBAC4(3) fluorescence intensity in 50 individual cells as shown in panel A. **p < 0.01.
**Figure S5.** Representative recordings showing that application of serum induced initial strong Ca^2+^ response, followed by Ca^2+^ oscillation.

## Data Availability

The original data that support the findings of this study are available from the corresponding authors upon reasonable request.
