# Supplementary material for: Functional cooperation between IKCa and TRPC1 channels regulates serum‐induced vascular smooth muscle cell proliferation via mediating Ca2+ influx and ERK1/2 activation
Source: Cell Prolif. 2022 Dec 23;56(4):e13385. doi: 10.1111/cpr.13385 (PMC10068941; doi:10.1111/cpr.13385)
Supplement: Supplementary file 1 — Figure S1. The representative image showing the siRNA‐transfected cells. siRNA was labelled with Cy3 (red colour). The percentage of red‐stained cells suggests that almost a majority of cells were successfully transfected. Figure S2. Serum induced an increase in the PCNA expression in VSMC. Representative western blots (top) and summary of data from three experiments (bottom) showing PCNA expression in cells cultured in the absence and presence of 10% FBS. **p < 0.01. Figure S3. ERK inhibition with PD98059 decreased the PCNA expression. Representative western blots (top) and summary of data from three experiments (bottom) showing PCNA expression in cells cultured in presence of 10% FBS without or with treatment with 5 μM PD98059. **p < 0.01. Figure S4. IKCa channel activation by 1‐EBIO results in membrane hyperpolarization in VSMC. (a) Representative recordings showing addition of 1‐EBIO induced an increase in DiBAC4(3) fluorescence intensity in cells transfected with siIKCa, but not in cells transfected with siCTL or siTRPC1. (b) Summary of relative DiBAC4(3) fluorescence intensity in 50 individual cells as shown in panel A. **p < 0.01. Figure S5. Representative recordings showing that application of serum induced initial strong Ca2+ response, followed by Ca2+ oscillation. [file CPR-56-e13385-s001.docx]

**
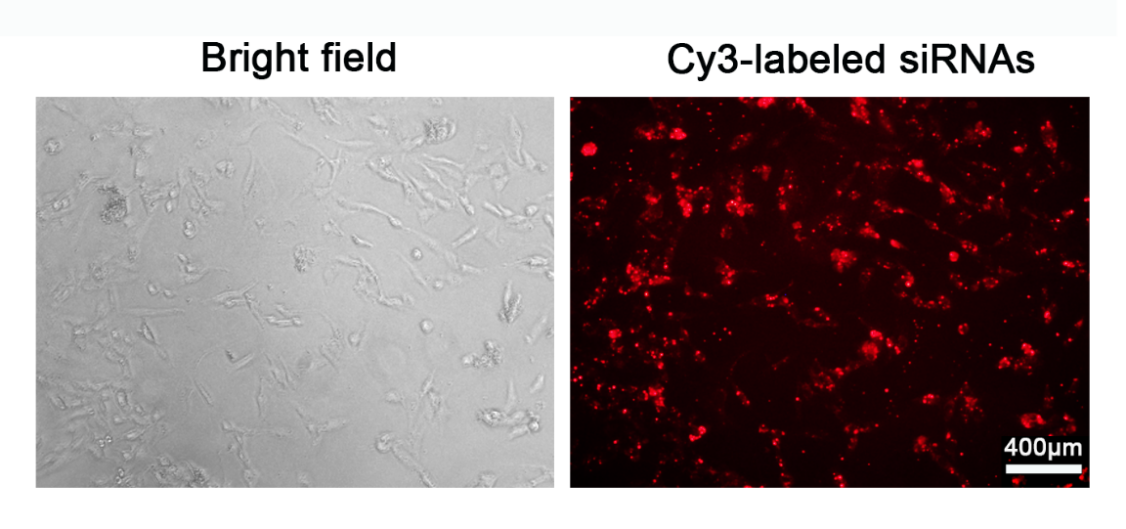
**

**Figure S1 The representative image showing the siRNA-transfected cells. siRNA was labeled with Cy3 (red color).** The percentage of red-stained cells suggests that almost a majority of cells were successfully transfected.


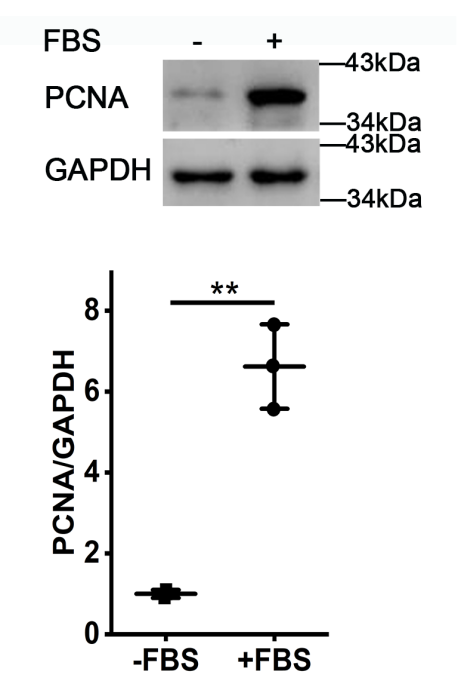


**Figure S2 Serum induced an increase in the PCNA expression in VSMC**. Representative western blots (top) and summary of data from three experiments (bottom) showing PCNA expression in cells cultured in the absence and presence of 10% FBS. **p < 0.01.


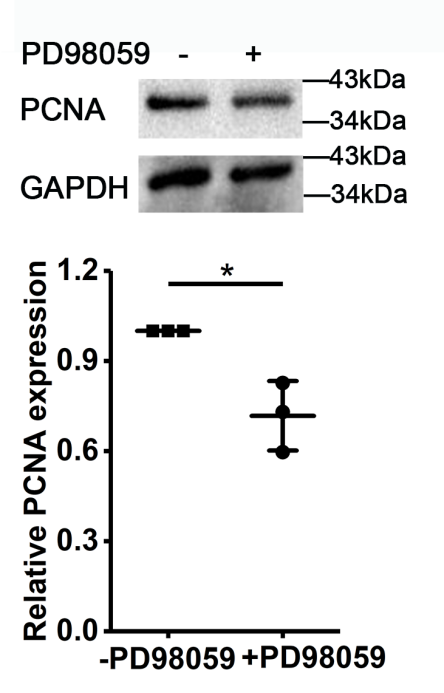


**Figure S3 ERK inhibition with PD98059 decreased the PCNA expression.** Representative western blots (top) and summary of data from three experiments (bottom) showing PCNA expression in cells cultured in presence of 10% FBS without or with treatment with 5 μM PD98059. ***p* < .01.


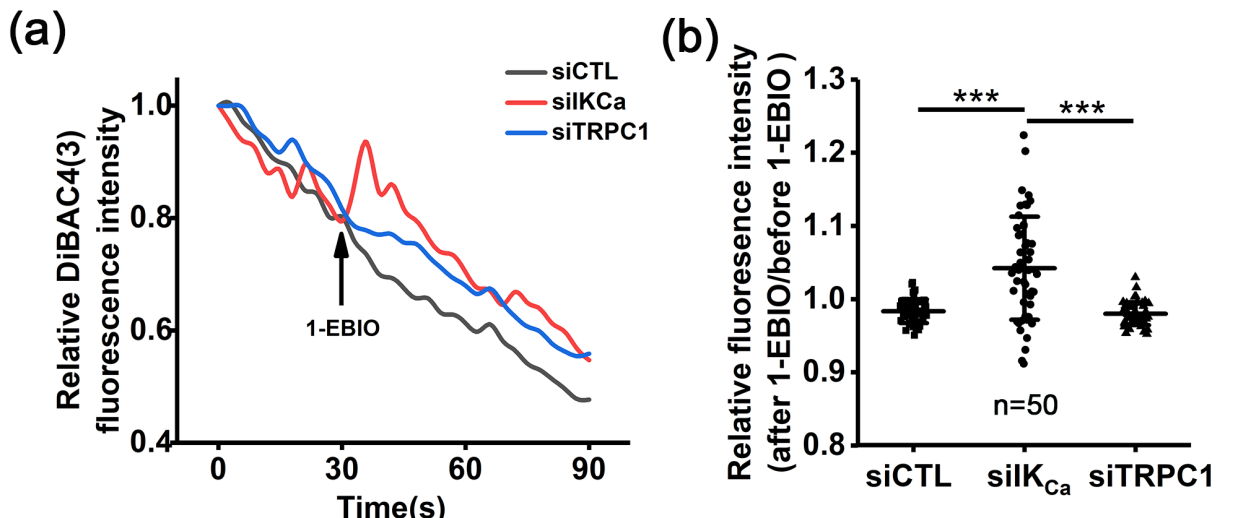


**Figure S4 IK_Ca_ channel activation by 1-EBIO results in membrane hyperpolarization in VSMC.** **(a)** Representative recordings showing addition of 1-EBIO induced an increase in DiBAC4(3) fluorescence intensity in cells transfected with siIK_Ca_, but not in cells transfected with siCTL or siTRPC1. **(b)** Summary of relative DiBAC4(3) fluorescence intensity in 50 individual cells as shown in panel A. ***p* < .01.





**Figure S5 Representative recordings showing that application of serum induced initial strong Ca^2+^ response, followed by Ca^2+^ oscillation.**
